# Application of Guanidine Hcl to Improve Enantioseparation of a Model Basic Drug, Cetirizine, By Capillary Electrophoresis Using Sulfated Β-Cyclodextrin

**Published:** 2018

**Authors:** Alireza Shafaati, Afshin Zarghi, Farin Sattary Javid

**Affiliations:** *Pharmaceutical Chemistry Depratment, School of Pharmacy, Shahid Beheshti University of Medical Sciences, Tehran, Iran.*

**Keywords:** Cetirizine, Chiral separation, Capillary electrophoresis, Guanidine HCl, Sulfated β-Cyclodextrin

## Abstract

A common approach in resolving enantiomers of chiral basic drugs by capillary electrophoresis (CE) is to use cyclodextrins (especially their anionic derivatives) as chiral selector in the acidic buffer (pH ≤ 3) in normal or reversed (carrier) mode. Then, some organic modifiers are added to the buffer solution if the resolution is not satisfactory. In case of cetirizine (CTN), applying the same approach, *i.e. *a reversed mode capillary zone electrophoresis (CZE) method with an acidic buffer and sulfated-β-cyclodextrine (S-bCD) as chiral selector, was failed and no complete enantioseparation was achieved. Different organic modifiers, like urea and triethylamine HCl, were used to improve chiral resolution which led to partial resolution of the two peaks. Then, guanidine HCl at a concnetration of 100 mM was added to the running buffer and an acceptable resolution of the enantiomers of the drug was obtained. The method was successfully applied to determine optical purity of a *levo*-cetirizine (l-CTN) sample.

## Introduction

The control of chiral purity of drug substances and pharmacokinetic studies of new chiral medicines places increasing demands on the development of analytical methods in the pharmaceutical field. Capillaryelectrophoresis (CE) has been found a powerful technique in chiral separations due to its versatility, high efficiency, short analysis times and lower cost of analysis (1, 2). Enantioseparations in this technique is accomplished by adding a chiral selector to the background electrolyte. Among many different kinds of selectors, cyclodextrins (CDs) and their derivatives continue as the most widely used chiral resolving agents (3, 4). The mechanism of chiral separation using CDs is based on different inclusion of the hydrophobic part of the enantiomers into the CD cavity, the stereoselectivity is enhanced by secondary interactions between the functional groups of the analyte with the hydroxyl (or derivetized hydroxyl) groups on the outside rim of the CD (4).

It is reported in the literature that negatively charged cyclodextines, especially sulfated β-CD (S-bCD) have been contributed significantly for the separation of basic drug enatiomers (4). Basically, they can be used as selectors in CE in normal polarity at low concentrations, but it is observed that application of this selector at low pH in reversed-polarity (carrier) mode can achieve the appropriate resolutions in enantioseparation studies of some basic compounds with high complexing capacity towards the S-bCD (4-6).

In the challenge of developing a separation method by CE, a variety of additives may be added to the buffer to improve the separation. One of the most important additives are organic modifiers which have important effect on resolutions, electrophoretic mobility, and migration time by modifying partition coefficients, the viscosity, and polarity of the background electrolyte, and the EOF as well (7). Application of organic solvents (such as acetornitirle and methanol) along with the chiral selectors is also investigated in chiral separation studies. For example, in the study by Ann Van Eeckhaut *et al*. acetonitrile is used to enhance the resolution of cetirizine enantiomers (8). 

Application of triethylamine as a cationic additive in the separation of peptides could optimize the resolution by blocking the negatively charged silanol groups on the capillary wall and reducing wall interactions between the positively charged peptides (at low pH) and the ionized silanol on the capillary wall (9). Urea is another additive typically used as a solubilizing agent due to the low aqueous solubility of β-CD in the concentration of 2M (10), but this agent can be used more widely due to its effect on complexation behavior. In the chiral separation of polychlorinated biphenyls using cyclodextrine derivatives, addition of 2M urea to the buffer solution was crucial to achieve the chiral separation (11). But, besides the usefulness of this additive, high concentrations of urea can make problems, as urea solutions are prone to crystallization and necessitating frequent cleaning of instruments (12).

**Figure 1:**
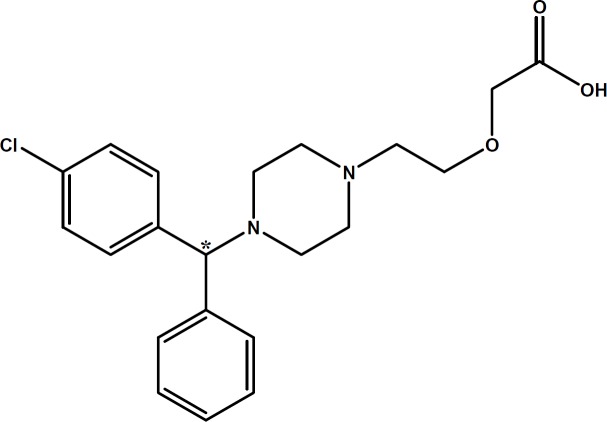
Chemical structure of cetirizine (CTN). Chiral center is marked with asterisk

In the course of our studies on chiral separation of basic drugs (13-15), cetirizine (CTN) (see Figure 1) was selected as a model basic drug for chiral separation using CE. CTN is a widely used second-generation histamine receptor antagonist, due to fewer central anticholinergic side-effects such as dry mouth and sedation (16). CTN is a racemate, consisting of equal quantities of two enantiomers; *levo*-cetirizine (R-enantiomer) and *dextro-*cetirizine (S-enantiomer). *In-vitro* studies using histamine-induced cutaneous and nasal responses have shown that *levo*-cetirizine is the more active enantiomer (17).

**Figure 2:**
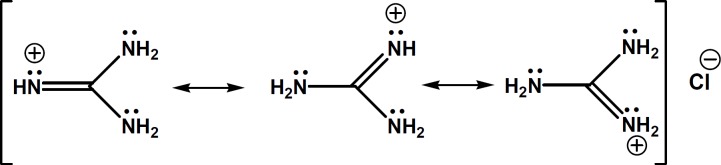
Chemical structure of guanidine HCl and its delocalized  -electrons and positive charge

To achieve appropriate separation of CTN enantiomers by CE using S-bCD as chiral selector, additives like organic solvents, urea and triethylamine were used to improve separation, but no baseline resolution was achieved. The aim of this work is to introduce guanidine HCl (GU) as an alternative buffer additive to urea and triethylamine, and to demonstarte its capabilities in chiral separation of CTN by CE. GU resembles to urea in structure (see Figure 2), but is cationic in aqueous solutions similar to triethylamine (18).

## Expeimental

*Chemicals*

Cetirizie HCl and *levo*-cetirizine (*levo*-CTN or –CTN) was kindly gifted by Noor Research and Education Institute (Tehran, Iran), sulfated beta-cyclodextrin were purchased from Sigma-AldrichChemie, GmbH (Germany). All other chemicals were of analytical grade reagents and purchased from Merck (Darmstadt, Germany). Deionized water was used in the preparation of all sample, rinsing, and buffer solutions.

*CE System*

All experiments were performed on a Prince-C650 CE system (Emmen, the Netherlands) equipped with UV/visible detector BISCHOFF (lambda 1010). CE analyses were performed using untreated fused-silica capillary of 50 μm internal diameter and 75-cm total length (25-cm effective length). The electrophoretic integration was performed by Dax Data Acquisition Analysis software (version 8.0) (Eindhoven, The Netherlands). The capillary was conditioned prior to the first use by rinsing with 1M NaOH for 15 min, followed by 0.1 M NaOH and water for 5 min each. Between runs, the capillary was flushed for 3 min with running buffer to guarantee good reproducibility.

*Electrophoretic separation condition*

The voltage applied was -15 kV at 25 °C, and the detection wavelength was set at 230 nm. Samples were introduced into the capillary by hydrodynamic injection for 6 sec at 50 mbar. The running buffer was a phosphate buffer at the studied pH values, containing S-bCD as the chiral selector and additives, which were freshly prepared daily and filtered through a 0.45 µm filter membrane. 

*Standards and sample solutions*

Standard stock solutions of the racemic and R-enantiomer of CTN, or *levo*-CTN, were prepared in water at a concentration of 1 mg/mL. Before injection, the stock solutions were diluted with water. All samples were injected in triplicate. A spiked solution of racemic cetirizine by *levo*-CTN was made to assign the higher peak as levocetirizine. All sample solutions were filtered through a 0.45 µm filter membrane prior to injection.

## Results and Discussion

In order to separate optical isomers by CE, it is necessary to introduce a chiral selector into the running buffer. Cyclodextrins (CD’s), native or modified, are the most popular chiral selectors used in CE (19). The use of chiral selectors carrying a charge opposite to that of the analytes can greatly improve the mobility difference between the two enantiomers (20, 21). As part of our studies on separation of enantiomers of chiral basic drugs (13-16), we tried to develop a method for enantioseparation of CTN, based on CE using S-bCD as chiral selector.

**Table 1:** Performance of the chiral CZE method on enantioseparation of cetirizine (CTN

	**Migration time (min)**		**Number of theoretical** **plates**	
	***l-CTN***	***d-CTN***	***l-CTN***	***d-CTN***
Within-day repeatability^a^	16.3 (5.1) c	17.4 (4.4)	16000 (6.3)	16600 (5.9)
Between-day repeatability^b^	16.2 (8.7)	17.3 (7.9)	15700 (7.7)	16300 (7.3)

Experimental conditions: as in Figure 5.

a Within-day repeatability: results from 10 experiments performed on the same day.

b Between-day repeatability: results from 6 experiments performed on 6 days.

c In parentheses %RSD.

**Figure 3:**
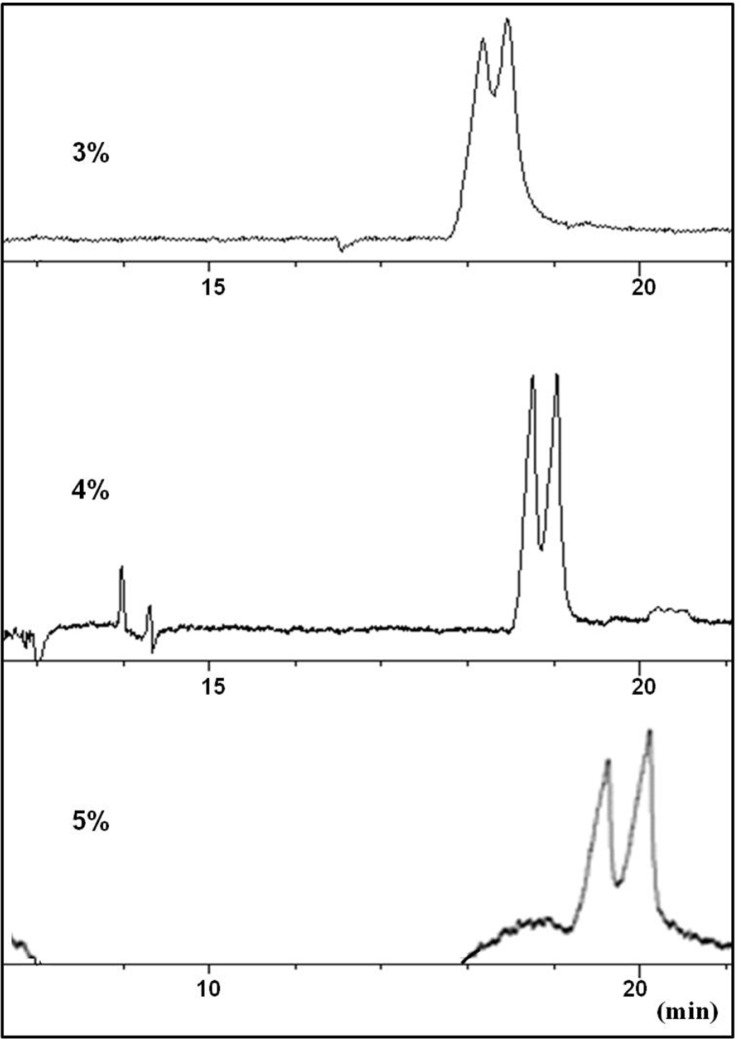
Effect of urea concentration (3-5% w/v) in the running buffer on the resolution of CTN enantiomers in a carrier (reverse mode) CZE method. Conditions as described in the text

**Figure 4:**
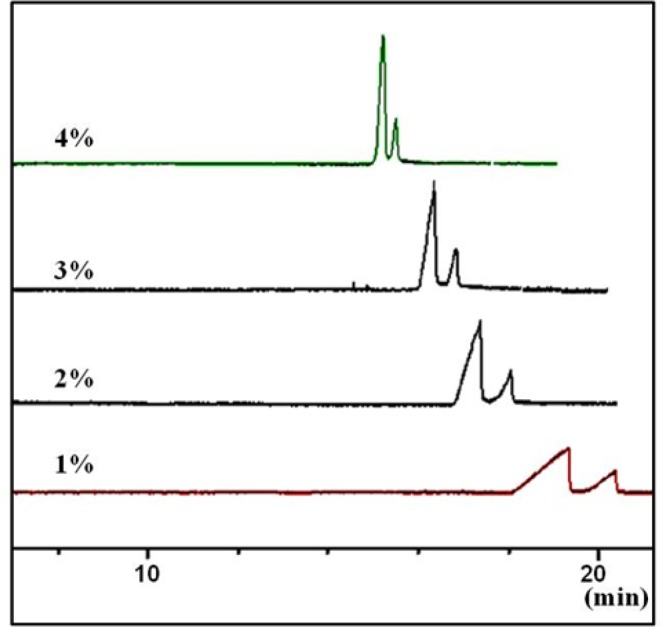
Effect of concentration of the chiral selector (sulfated  -CD) on peak shape and resolutions of the two enantiomers of CTN in a carrier (reverse mode) CZE method. Conditions as described in the tex

**Figure 5:**
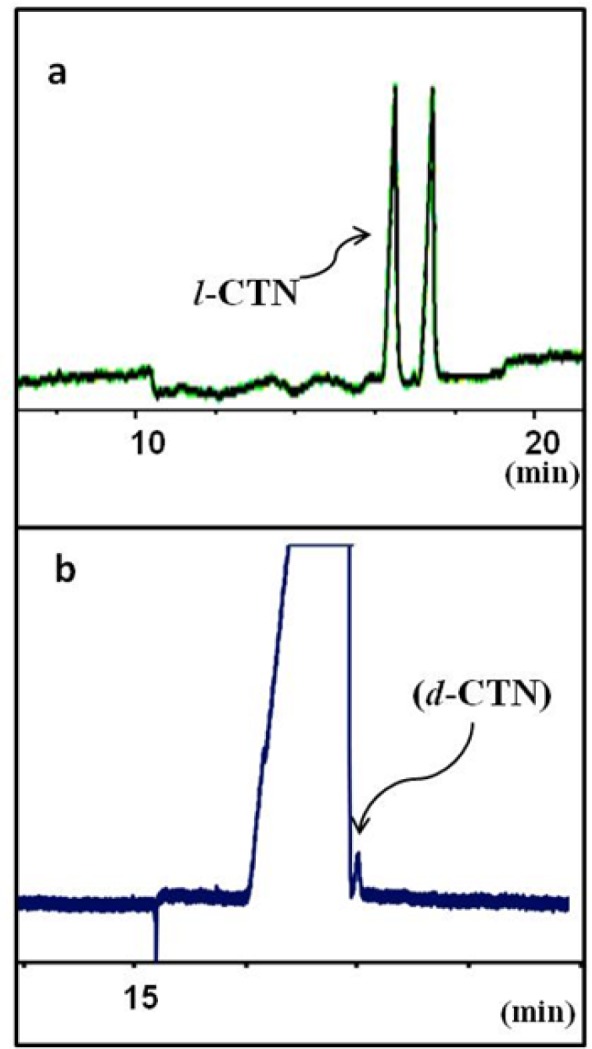
Separation of *levo*-cetirizin (*l*-CTN) and *dextro*-cetirizine (*d*-CTN) under optimum CZE conditions: running buffer contains guanidine HCl 100 mM, sulfated-beta CD 3% w/v in 25 mM phosphate buffer at pH 3.0 and the applied voltage was -15 kV. (a) a solution of *rac*-cetirizine and (b) a sample of pure *levo* enantiomer (*l*-CTN) spiked with 0.01% w/w *dextro* isomer (*d*-CTN

*Separation in the presence of sulfated β-CD*

As shown in Figure 1, CTN possesses three ionizable functional groups including an acidic group with p*K*a = 2.9 (carboxylic group), a relatively stronger basic group with p*K*a = 8.0 and a second weaker basic group (p*K*a = 2.2) (22). In the enantioseparation of CTN by Mikuš (23), the EOF is suppressed by a polymer coating and the separation method is applied in higher pH. This may cause weaker positive nitrogen along with negative carboxylic acid groups which helped to achieve the separation by highly derivatized CDs. In an another study (24) for separation of CTN enentiomers, S-bCD was applied at concentration of 1% w/v, with the pH of buffer that was optimized at 8.7. Under the specified conditions, the anionic CD and anionic species of CTN, repelled each other which resulted in a successful enantioselective inclusion. Adjusting the exact optimized buffer pH is very crucial in this technique. Also, in higher pH solutions, non-selective electroosmotic flow must be eliminated by using coated capillaries

In this study, a carrier-mode CE separation (14) using an acidic pH buffer containing negatively charged beta CD (S-bCD) was proposed based on the results found in the literature on the chiral separation of basic drugs (25) and the results of our previous work on enantiosepation of amlodipine (14, 15). Although, adsorption of the cationic CTN on the inner wall of the capillary must be considered, lowering pH of the buffer to 3 or even less will greatly reduce the chance of adsorption of the analyte, due to the minimum ionization of the silanol groups on the capillary wall. Application of sulfated CD derivatives was suggested as an analyte carrier for effective enantioseparation of CTN. In higher pH buffers, the electroosmotic force which is against the carrier movement direction causes broad peaks. Therefore, a CZE method with reversed polarity at low pH buffer was proposed for the separation.

Different concentrations of S-bCD (1-5% w/v) were examined for the separation of CTN using phoasphate buffer pH 3. In this pH, at any concentrations of the selector, no baseline separation was achieved; suggesting that a strong interaction between the selector and CTN (which has two basic sites) may prohibit the enantioselectivity of the selector and subsequent baseline resolution achievement in acidic pH. Under these conditions, the separation of the enantiomers is typically based on inclusion complexation along with extra stabilization with electrostatic interaction between the positively charged nitrogens of the analyte and the negatively charged sulfate group of the CD (26). But, due to strong electrostatic interactions of cationic molecules like CTN with negatively charged cyclodextrines, the migration of the cation was retarded even at low concentrations of the selector used (27). Thus, we considered a suitable buffer additive to optimize the balance between the enantioseparative inclusion and the ionic interactions between the selector and the basic drug. As a result, adding an additive to BGE to reduce secondary electrostatic interactions between CTN and the selector was suggested to obtain baseline separation of the enantiomers.

*Applications of buffer additives*

In oreder to improve the resolution of CTN enantiomers, addition of widely used organic modifiers like methanol and acetonitrile in the concentration range of 5 to 30% v/v to the running buffer were examined. But peak broadening with no resolution was observed.

At this stage, urea was considered as an additive to modify the complexation between the selector and CTN. Partial resolution of CTN enantiomers was observed by adding urea at concentration levels of 3-4 M to the buffer (Figure 3). Applying higher concentrations of urea resulted in peak broadening with no improvement in the resolution of the enantiomers (Figure 3). It seems that urea was partially able to influence on the strong interaction between the selector and CTN, but could not modify the interaction in favor of chiral recognition. Higher urea concentration led to peak broadening which was possibly due to unwanted interaction of the weakly basic urea with the selectorl-CTN complex which, in turn, reduced electrophoretic mobility of both selector-enantiomer complexes. Although, higher viscosity of the buffer solutions resulted from a significant amount of urea must also be considered.

In the next step, addition of a modifier like triethylamine (p*Ka* = 10.7), which is readily protonated in acidic solution was considered which supposed to strongly affect the binding of the cationic enantiomers with the negatively charged selector. No resolution was observed at any attempted concentration of triethylamine. Peak broadening and longer migration time of CTN peak was attributed to a competition between CTN and triethylyamine to bind to the selector, in which triethylamine was succeeded due to prior equilibration with the buffer. 

In search of a modifier with stronger affinity than urea to bind to sulfated β-CD but weaker than triethylamine, we came to guanidine HCl, in which the molecule bears a single positive charge (similar to triethylamine), but the charge is not localized on a specific atom and disperses over the whole molecule (28). In fact, in guanidinium species, the nitrogen electron pairs are involved in intramolecular π-bonding and the energy of this π-system is lowered by positive charge. Thus, guanidinium ion shows a negligible ability to behave as a Lewis base. The exceptionally high basicity of guanidine (p*Ka* = 13.6) is suggested to be associated with a form of aromaticity and delocalization of the six π-electrons across the symmetric Y-shaped CN3 unit in the guanidinium cation, and the term Y-aromaticity has been used to describe this phenomenon (see Figure 2) (28).

Guanidine is different from urea in terms of their basicity and nucleophilicity and also in their ability to make hydrogen bonds. It is shown that urea H-bonds to peptide NH groups and probably also to peptide CO groups to a degree that is able to explain the thermodynamic effect of urea on the protein backbone, but guanidine does not H-bond to the peptide group. Thus, urea, but not guanidinium, destabilizes proteins by forming hydrogen bonds to the peptide group (29). In our case, the ability of urea to make H-bonds may be responsible in formation of weak and transient binding of urea to the selector which is readily displaced by CTN enantiomers and make stronger electrostatic bonds with sulfate groups on the selector. Displacement of urea with enantiomers may partially, but not completely, promote chiral recognition of the two enantiomers. In case of guanidinium ions, the interaction with anionic sulfate groups on the selector is stronger than urea, but weaker than triethylamine and therefore, their displacement with CTN enantiomers may provide proper conditions for chiral recognitions of the two enantiomers.

*Optimum separation conditions*

Resolution of CTN enantiomers were started by adding 40 mM guanidine HCl to the buffer containing a constant concentration of 3% w/v of S-bCD , but optimum resolution was observed at 100 mM of the additive. Higher concentration of guanidine would result in increased buffer ionic concentration and risk of Joule heating.

Based on our previous experiences with S-bCD (14), concentration of the chiral selector at 0.1-4.0% w/v in the BGE were tested, at a constant guanine HCl concentration of 100 mM. In the lower concentrations than 1% w/v of the selector, the broad peaks were seen. On the other hand, at the concentrations higher than 3% w/v, the sharp peaks were obtained in the cost of lower resolution (Figure 4). Thus, concentration of the selector was set at 3% w/v.

To study the influence of the pH, buffers with pH values of 2.5 to 4.0 were attempted while the other parameters were kept constant. In lower pH the peaks are sharper with the shorter migration time, but lower resolution factor was obtained. This can be attributed to the stronger positive charge of the analyte and the stronger interactions with the carrier and also the weaker electroosmotic flow force. On the other hand, as the pH of the running buffer was raised, the peaks were broadened, which is attributed to the opposite direction of the EOF and the direction of the carrier movement. As the result, pH 3 as selected as the optimum buffer pH.

Also, the influence of phosphate buffer (pH 3) concentration on the resolution of the separtion was studied in the range 10–50 mM maintaining the concentration selector at 3% w/v and guanidin HCl at 100 mM. It was concluded that although higher buffer concentrations led to better peak shapes, buffer concentrations more than 25 mM would also led to Joule heating and baseline aberrations.

Figure 5a showes the final electropherogram of the optimized background electrolyte composition as guanidine HCl 100 mM, S-bCD 3% w/v in 25 mM phosphate buffer at pH 3.0. The applied voltage was -15 kV. Figure 5b shows application of the optimized method to the determination of real *levo-*CTN sample spiked with *dextero*-CTN at an accepted maximum limit. 

*Method Validation*

The proposed CE method was partially validated using the ICH guidelines (30). The specificity of the method was assessed by injecting a solution prepared from a mixture of *rac*-CTN and *levo*-CTN. The limit of quantification (LOQ) for *dextro*-CTN was found less than 0.1% w/w, which is maximum allowable limit of the compound as an impurity in pure *levo*-CTN (Figure 5). Other validation parameters are summarized in Table 1.

## Conclusions

It is clear CTN like other basic molecules, acquires positive charge in acidic solutions. Therefore, its interaction with the oppositely charged chiral selectors is more likely to occure. But, at the same time, a strong interaction between the selector and CTN, which has two basic sites, may prohibit the enantioselectivity of the selector and subsequent baseline resolution achievement in acidic solutions. Guanidine HCl was used to modify interaction of the analyte with the selector in favor of chiral separation. Thus, guanidine HCl is introduced as an alternative to both urea and triethylamine HCl as modifiers in chiral resolution of the basic molecules. 
